# The Dutch version of the Oral Health Impact Profile (OHIP-NL): Translation, reliability and construct validity

**DOI:** 10.1186/1472-6831-8-11

**Published:** 2008-04-11

**Authors:** Marylee J van der Meulen, Mike T John, Machiel Naeije, Frank Lobbezoo

**Affiliations:** 1 Department of Oral Function, Academic Centre for Dentistry Amsterdam (ACTA), Louwesweg 1, 1066 EA, Amsterdam, The Netherlands; 2 Centre for Special Dental Care (SBT), Amsterdam, The Netherlands; 3 Department of Prosthodontics and Material Science, University of Leipzig, Germany

## Abstract

**Background:**

The purpose of this study was to make a cross-culturally adapted, Dutch version of the Oral Health Impact Profile (OHIP), a 49-item questionnaire measuring oral health-related quality of life, and to examine its psychometric properties.

**Methods:**

The original English version of the OHIP was translated into the Dutch language, following the guidelines for cross-cultural adaptation of health-related quality of life measures. The resulting OHIP-NL's psychometric properties were examined in a sample of 119 patients (68.9 % women; mean age = 57.1 ± 12.2 yrs). They were referred to the clinic of Prosthodontics and Implantology with complaints concerning their partial or full dentures or other problems with missing teeth. To establish the reliability of the OHIP-NL, internal consistency and test-retest reliability (N = 41; 1 – 2 weeks interval) were examined, using Cronbach's alpha and intraclass correlation coefficients (ICC), respectively. Further, construct validity was established by calculating ANOVA.

**Results:**

Internal consistency and test-retest reliability were excellent (Cronbach's alpha = 0.82 – 0.97; ICC = 0.78 – 0.90). In addition, all associations were significant and in the expected direction.

**Conclusion:**

In conclusion: the OHIP-NL can be considered a reliable and valid instrument to measure oral health-related quality of life.

## Background

Since the recognition of the multidimensional character of health issues, a conceptual framework has been created to analyze the role of psychosocial factors in health and disease [1]. In order to study the role of such factors in dentistry, Reisine *et al*. [2] examined dental patients with the use of a general health-related quality of life measure, the Sickness Impact Profile. Specific instruments to measure the impact of oral disease on the quality of life of individuals were developed as well, like the Social Impact of Dental Disease [3] and the Dental Impact Profile [4]. Likewise, Slade & Spencer [5] published a study on the development and evaluation of the Oral Health Impact Profile (OHIP), in which the guidelines of the World Health Organization [6], to distinguish more systematically between functional limitation and social impact of physical problems, were followed. This instrument consists of 49 questions about the negative effects of oral conditions on daily functioning. It was shown to be a reliable and valid instrument for the examination of oral disease-related disability in different patient groups [7-12].

To be able to assess levels of oral health-related quality of life in non-English-speaking populations, cross-culturally adapted translations of the OHIP-E (i.e., the original English-language version of the 49-item OHIP) have already been accomplished in several countries [13-17]. The comparison between levels of perceived oral health-related quality of life in The Netherlands and those in other countries and cultures demanded the development of a cross-culturally adapted Dutch version of the OHIP-E. Therefore, the aim of this study was to translate the OHIP-E into the Dutch language, and to examine the reliability and construct validity of the resulting OHIP-NL.

## Methods

### Oral Health Impact Profile

The English-language Oral Health Impact Profile (OHIP-E) consists of 49 questions that are conceptually categorized according to Locker's model [1]. This model uses a hierarchy in which different levels of disease-related disruptive impairment are distinguished, resulting in seven domains (see left column in Table 1). The domain 'Functional limitation' (9 questions) concerns the loss of function of parts of the body, like difficulty with chewing. The domains 'Physical discomfort' (9 questions) and 'Psychological discomfort' (5 questions) deal with experiences of pain and discomfort, such as toothache and feeling miserable. The domains 'Physical disability' (9 questions), 'Psychological disability' (6 questions), and 'Social disability' (5 questions) refer to limitations in performing daily life activities, like avoiding certain foods, lack of concentration, and feeling irritable with others, respectively. Finally, the domain 'Handicap' (6 questions) concerns a sense of disadvantage in functioning, like suffering financial loss due to dental problems. Answers to the 49 questions are scored on 5-point ordinal scales, ranging from never (0), hardly ever (1), occasionally (2), and fairly often (3), to very often (4). The 49 scale scores are then summed; the total score can thus range from 0 to 196. Similarly, domain scores can be obtained. Higher scores imply a more impaired oral health-related quality of life.

**Table 1:** Mean total scores and mean item scores of the total OHIP-NL and of its seven domains (the number of constituent questions is given between parentheses)

	Mean total score	SD	Mean item score	SD
Total OHIP-NL (49)	44.1	40.2	0.9	0.8
Functional limitation (9)	10.6	7.8	1.2	0.9
Physical discomfort (9)	9.2	8.1	1.0	0.9
Psychological discomfort (5)	6.6	5.9	1.3	1.2
Physical disability (9)	7.2	8.5	0.8	1.0
Psychological disability (6)	4.8	6.1	0.8	1.0
Social disability (5)	2.6	4.9	0.5	1.0
Handicap (6)	3.2	5.4	0.5	0.9

### Translation into Dutch

The OHIP-E was translated into Dutch by four different translators through the use of the so-called forward-backward approach, thereby mostly following the guidelines for cross-cultural adaptation of health-related quality of life measures [18,19]. The forward translation into Dutch was performed by two independent, bilingual translators whose native language was Dutch. One of them was an expert in quality of life measures; the other had no specific experience in that field. The two forward translations were compared and synthesized into one common version by an expert panel, consisting of a dentist/TMD-expert and a psychologist, specialized in the field of dentistry. When competing options for a translation were debated, other bilingual experts were consulted. The resulting common forward translation was translated back into English by two independent, professional translators whose native language was English. The two back-translations were discussed again by the expert panel, comparing semantic, experiential, and conceptual equivalence between the two versions. The expert panel then reviewed the back-translations against the original OHIP-E. Finally, the resulting OHIP-NL, that uses a 1-month reference period (see Discussion), was read and commented upon by members of the Department of Oral Function. An electronic version of the OHIP-NL can be obtained from the authors, free of charge.

### Study sample and procedure

In order to study the reliability and construct validity of the OHIP-NL, a convenience sample of 119 consecutive patients (68.9 % women; mean age ± SD = 57.1 ± 12.2 yrs) was recruited during a pre-specified period of 4 months. They were referred by their dentists to the clinic of Prosthodontics and Implantology of the Department of Oral Function, ACTA, with complaints concerning their dentures or other problems with missing teeth. The purpose of the referral was either to repair or replace their dental provisions and/or to undergo implant surgery. All patients signed a statement of informed consent. Immediately after the patients registered at the clinic, they were sent the OHIP-NL and a dental complaints questionnaire, concerning measures that were used to assess the construct validity of the OHIP-NL. They completed both questionnaires at home and returned them by mail.

### Internal consistency

Internal consistency of the total OHIP-NL as well as of its seven constituent domains was assessed by calculating Cronbach's alphas. According to Bland & Altman [20], Cronbach's alphas of 0.70 – 0.80 are considered satisfactory for a reliable comparison between groups. However, for clinical purposes, a minimum of 0.90 is required, while values of at least 0.95 are considered desirable [20]. In addition, inter-item correlations were calculated, as to register a possible increase of the Cronbach's alphas due to the length of the questionnaire [21]. According to Clark & Watson [22], a mean inter-item correlation of 0.15 – 0.20 is desirable for scales that measure broad characteristics, while values of 0.40–0.50 are required for scales tapping narrower ones, which is the case in the present study.

### Test-retest reliability

A convenience sub-sample of 41 consecutive patients received a second OHIP-NL immediately after their first completed questionnaire was received by mail. The interval between the first and second instrument administration was one to two weeks. Test-retest reliability of the OHIP-NL and of its seven domains was assessed by calculating intraclass correlation coefficients (ICCs). ICC values were based on the outcome of a one-way repeated measures ANOVA [23] and on the mean differences between the two trials. Following the method of Bland & Altman [24], which involves the computation of the standard deviation of the differences between the measures at the two time points, limits of agreement around the mean difference were calculated as 1.96 times the standard deviation of the differences. Hence, the limits of agreement represent the test-retest differences that can be expected for 95% of the individuals in the sample. ICCs were interpreted according to Fleiss [25]: ICC < 0.40 = poor reliability; ICC ≥ 0.40 but ICC ≤ 0.75 = fair to good reliability; and ICC > 0.75 = excellent reliability.

### Construct validity

In order to establish construct validity of the OHIP-NL, aspects of both convergent validity and group validity were investigated. For convergent validity, the relationships were examined between OHIP-NL scores and other measures that are assumed to be derived from the same construct. Variables used for this analysis were self-reported oral health status, and complaints-related disability. It was hypothesized that low oral status and high complaints-related disability would be associated with high scores on the OHIP-NL. For group validity, OHIP-NL scores were compared between groups of patients who either had or didn't have certain dental conditions that have shown to be related to oral health-related quality of life [12,13,17,26]. It was expected that a high number of missing teeth, having a partial or full denture, and more burning mouth- and other dental complaints, would be related to high OHIP-NL scores. Using the statistical software package STATA 9.0 (StataCorp. 2005, Stata Statistical Software, College Station, TX, USA) and with the probability of a type I error set at the 0.05 level, ANOVA was used for convergent and group validity; omega2 was calculated to measure the strength of the associations [27]. Omega2 < 0.01 is considered to be a small, 0.06 a medium, and 0.14 a large association; effect size of 0.10 is considered to be small, 0.25 medium and 0.40 large. Associations were examined between the mean total OHIP-NL score and:

1. Oral health status – *"Would you say your oral health in general is...?"*, to which patients could reply with the following options: excellent (1), very good (2), good (3), fair (4), or poor (5).

2. Burning mouth syndrome – *"Do you sometimes have a burning feeling in your mouth?"*. This could be answered as 'No' or 'Yes'.

3. Dental status – *"Do you have a natural dentition, a partial denture, or a full denture?"*.

4. Number of teeth still present in the upper and lower jaw – "*How many teeth do you have in your upper/lower jaw?"*. The response could range from 0 to 32.

5. Specific complaints related to denture or missing teeth – *"Which dental complaints do you have at this moment?"*. Specifically, patients could indicate to what extent they were bothered by the following 12 possible complaints: gagging, pain, pressure, burning feeling, aesthetics, looseness of fit, difficulty with chewing, difficulty with talking, dry mouth, wet mouth, painful corners of mouth, or other complaints (this latter option was used by less than 10 % of the patients). The answers could range from none (1), a little bit (2), somewhat (3), and rather much (4), to very much (5). The total score could thus range from 12 to 60.

6. Dental complaints-related disability – *"Do your dental complaints have a negative impact on ...your mood? ...your physical/psychological health? ...your work? ...your relations? ...your lovemaking?"*. Responses, on 5-point scales, ranged from none (1), a little bit (2), somewhat (3), and rather much (4), to very much (5). The total score could thus range from 5 to 25.

For the associations 4 (number of teeth still present in the upper and lower jaw), 5 (specific complaints related to denture or missing teeth), and 6 (dental complaints-related disability), the total set of actually obtained scores was split into three groups (tertiles). In principle, every tertile contains an equal percentage of observations. However, since patients with the same score are grouped together in the same tertile, a relatively high frequency of a certain score sometimes caused a skewed distribution of patients over the tertiles.

### Control measures

In order to control for a possible tendency of patients to rate most dental questions in an identical way, another dental factor, which was expected *not *to be related to oral health-related quality of life, was used. For this purpose, self-reports of biting on nails, on pens, and on chewing gum were used, using the question *"How often have you engaged in the following activities during the past time?" *[28]. These oral habits were rated on a 5-point scale, ranging from never (0), sometimes (1), regularly (2), and often (3), to always (4). The total score could range from 0 to 12. As for the validity assessment, its association with OHIP-NL scores was calculated (see Construct validity).

### Missing data

Subjects, who missed more than five questions on the total OHIP-NL, or more than two questions from within one of the seven domains, were discarded. Missing answers that did not exceed these criteria, were imputed using regression imputation within the relevant domain, i.e., the domain's mean was calculated and entered for missing values.

## Results

No difficulties with semantic, experiential, or conceptual equivalence were encountered during any part of the translation procedure. Items for discussion were only related to finding idiomatic equivalences. Examples were expressions like 'painful aching', a tooth that doesn't 'look right', a 'sense of taste', and 'affected by', for which several translations are possible that would all be very well understandable by a Dutch speaking person.

No patients had to be discarded for missing more than five questions on the total OHIP-NL or more than two questions from within one of the seven domains. In seven patients, a total of 14 missing answers that did not exceed these criteria were imputed.

The mean total scores, and the mean item scores of the total OHIP-NL and of its seven constituent domains, are shown in Table 1; their internal consistency, measured as Cronbach's alpha's, in Table 2. Cronbach's alpha for the total OHIP-NL was 0.97 and fulfilled the criterion for clinical usefulness of the instrument. The average inter-item correlation of the total OHIP-NL was within the desired range of 0.40–0.50. For the seven domains, all Cronbach's alpha values exceeded the 0.80 threshold for being considered satisfactory for making group comparisons. Except for 'Functional limitation', 'Physical discomfort', and 'Physical disability', the Cronbach's alpha values also exceeded the 0.90 threshold for clinical applications. Five domains yielded a mean inter-item correlation value higher than 0.50, i.e., outside the desired range of 0.40–0.50, that is required for the reliable use of 'narrow' (i.e., specific) scales like the ones of the OHIP-NL. The intraclass correlation coefficients (ICCs), characterizing the test-retest reliability of the total OHIP-NL and of its seven constituent domains, are shown in Table 3. Although all ICC values could be qualified as excellent, the limits of agreement indicate a considerable variability for the individual test-retest differences, both for the total OHIP-NL and for its seven constituent domains.

**Table 2:** Internal consistency measured for the total OHIP-NL and for its seven domains: Cronbach's alphas and average inter-item correlations

	Cronbach's alpha	Average inter-item correlation
Total OHIP-NL	0.97	0.42
Functional limitation	0.82	0.34
Physical discomfort	0.87	0.42
Psychological discomfort	0.90	0.64
Physical disability	0.88	0.54
Psychological disability	0.92	0.66
Social disability	0.95	0.79
Handicap	0.92	0.65

**Table 3:** Test-retest reliability measured for the total OHIP-NL and for its seven domains: Intraclass correlation coefficients (ICCs), mean differences, and limits of agreement

	ICC	Mean difference	Limits of agreement
Total OHIP-NL	0.90	-5.6	-37.9 to 26.7
Functional limitation	0.89	-0.2	-7.6 to 7.3
Physical discomfort	0.78	-1.9	-11.3 to 7.5
Psychological discomfort	0.87	-6.1	-6.5 to 5.4
Physical disability	0.85	-0.8	-9.5 to 8.0
Psychological disability	0.86	-1.0	-6.9 to 4.9
Social disability	0.89	-0.6	-4.3 to 3.1
Handicap	0.89	-0.6	-5.4 to 4.1

The outcomes of the convergent and group validity assessments can be gathered from Table 4. Both measures used for convergent validity (oral health status, and complaints-related disability) were significant at the 0.001 level. The effect size was large for both variables. Except for 'Burning mouth syndrome', for which the F- value was low and did not reach statistical significance, all self-reported aspects of oral conditions, related to group validity, were significantly correlated with the total OHIP-NL scores (P < 0.01 – 0.001). The associations were all in the expected direction. For example, the F value for the association between the total OHIP-NL scores and the 'Number of teeth' (5.60; P < 0.01) indicates, that the less teeth are still present in the upper and lower jaw, the more impairment there is of the oral health-related quality of life. Likewise, the F-value for 'Specific dental complaints' (38.09; P < 0.001) indicates, that more complaints in relation to dentures or missing teeth are associated with a higher impairment. Effect sizes for group validity measures were medium (oral health status, and number of teeth), and high (specific dental complaints)

**Table 4:** Construct validity: associations between the mean total OHIP-NL scores and self-reported aspects of oral conditions, complaints, and disability and measure of strength of the associations (omega2)

		*n*	OHIP-NL	ANOVA F (df)	Omega2 (effect size)
Oral health status	Excellent	7	25.3	8.16*** (4)	0.19 (0.49)
	Very good	20	23.6		
	Good	45	39.4		
	Fair	34	49.7		
	Poor	12	93.5		
Burning mouth syndrome	No	95	40.7	3.44 NS (1)	0.02 (0.14)
	Yes	22	58.2		
Dental status	Natural dentition	37	27.0	4.90** (2)	0.06 (0.27)
	Partial denture	39	53.8		
	Full denture	35	47.2		
Number of teeth	1st tertile (<7)	41	56.1	5.60** (2)	0.07 (0.28)
	2nd tertile (7–21)	37	45.4		
	3rd tertile (>21)	37	27.0		
Specific dental complaints	1st tertile (<15)	40	20.7	38.09*** (2)	0.39 (0.80)
	2nd tertile (15–21)	40	33.7		
	3rd tertile (>21)	36	79.7		
Dental complaints-related disability	1st tertile (5)	49	19.4	50.19*** (2)	0.47 (0.93)
	2nd tertile (6–8)	27	36.7		
	3rd tertile (>8)	37	81.9		
Self-reported oral habits	Absent	81	43.3	0.00 NS (1)	-0.01 (0.00)
	Present	37	43.1		

NS = not significant; ** = P < 0.01; *** = P < 0.001

Self-reported oral habits (i.e., the control measures) were not related to the total OHIP-NL scores (Table 4). Because about two thirds of the patients scored three times 'never' on these control questions. It was therefore decided to dichotomize the answers as 'Absent' (total score = 0) and 'Present' (total score > 0).

## Discussion

In the present study, the original English-language Oral Health Impact Profile (OHIP-E) was translated into Dutch, mostly following the international guidelines for cross-cultural adaptation of self-reported measures [18,19]. The resulting OHIP-NL showed good psychometric properties: both its reliability and its construct validity were satisfactory. The new instrument is therefore suitable for use in multi-national and multi-cultural studies to self-perceived oral health-related quality of life.

Where the afore-mentioned international guidelines suggest a small-scale field test to be performed prior to the implementation of a new instrument [18,19], final testing of the OHIP-NL was done by discussions with colleagues of the Department of Oral Function and bilingual experts instead. Despite this minor departure from the recommended procedure, we have confidence in the quality of the translation process, because the discussions only yielded some small idiomatic issues while no difficulties with semantic, experiential, or conceptual equivalence were encountered. Further, no patients had to be discarded for omitting too many questions of the OHIP-NL, suggesting that the formulations were clearly understandable for Dutch-speaking individuals. Patients showed no signs of misunderstanding the questions or instructions of the OHIP-NL: only very few modifications or cross-outs of responses were found on the paper forms. This supported our impression that the questions were comprehensible, and that the cross-cultural adaptation of the OHIP-E had been accomplished successfully.

The patients' compliance was good. Even though the OHIP-NL consists of many questions, most patients seemed willing (or even eager) to make a statement about the perceived negative effects of their dental condition on their quality of life. The questionnaire's length thus seems not to be a factor that will interfere with the collection of the OHIP-NL data.

In some previous studies, so-called 'weighing' of the OHIP items was performed as to obtain a reflection of the relative importance of each question for the subject [3,13]. In the present study, full question weights were not determined or used, because they did not result in improvements of measurement properties in the recent study by John *et al*. [13]. To confirm or refute this latter finding, we did apply an indirect weighing technique to our data. This technique, recommended by Slade [29], consists of counting 'fairly often' and 'very often' responses only, implicating that these questions would be more important for the patient than questions scoring 'never', 'hardly ever', or 'occasionally'. The latter responses are then scored as '0'; the 'fairly often' and 'very often' responses, as '1'. This results in a count summary score that could range from 0 to 49. When applied to the present data, the Pearson correlation between the sum scores of all responses (i.e., the method used in this study) and the count summary scores was 0.95. (P = 0.00). Not unexpectedly, the outcomes of the data analyses using the count summary scores (results not shown) were therefore very similar to the sum scores of all responses. This corroborates the recommendation of John *et al*. [13] not to weigh OHIP items for most purposes.

In the original OHIP study (5), no specific reference period was recommended, although Slade & Spencer (5) do specify that all 49 questions should refer to a fixed time-period. Nevertheless, most studies so far do not mention the time span over which patients reported their oral health-related quality of life, with a few exceptions. Slade [29], for example, used a 12-month period for reference. John *et al*. [13] showed that a 1-month reference period yields the highest ICC values and the narrowest limits of agreement as compared to a 1-year period and lifetime experience. In other words: the 1-month reference period is characterized by the highest test-retest reliability. Further, this time span accomplishes a high responsiveness in treatment follow-up studies [13]. For those reasons, the 1-month reference period was chosen for the OHIP-NL.

Internal consistency of the total OHIP-NL and of its seven constituent domains fulfilled the criteria for group comparisons, while most of the domains also fulfilled the criteria for clinical applications [20]. It should be noted that very high Cronbach's alphas can be the result of a high number of items in a questionnaire. Therefore, a maximum alpha of 0.90 is sometimes recommended by other authors [21,22]. Especially when the average inter-item correlations are higher than 0.50, redundancy of questions can be suspected. Fortunately, the average inter-item correlation of the total OHIP-NL was within the desired range of 0.40–0.50. The average inter-item correlations of five of the seven separate domains however, were higher than 0.50, which may indicate a redundancy of questions within those domains. As stated in the introduction, the seven domains of the OHIP-E were obtained on a theoretical basis, instead of by factor analysis. In the Dutch translation, all questions of these original seven domains were adopted. Factor analysis of the OHIP-NL might confirm the outcome of a German study, which resulted in a selection of domains and questions that deviated from the OHIP-E [30]. Developing a shorter version of the OHIP-NL, may improve some of the statistical properties of the questionnaire.

Test-retest reliability of the total OHIP-NL as well as of its domains could be qualified as excellent. This is a common finding in other translated versions (viz., the German, Chinese, Swedish, Italian, and Hungarian ones), both in the 49-item versions and in the short 14-item versions, and regardless of the composition of the study sample (e.g., prosthodontic patients, TMD patients) [13-17,31].

In this study, special emphasis was placed on construct validity measures. All *a priori *hypothesized associations between the mean total OHIP-NL scores and self-reported aspects of oral conditions, complaints, and disability were confirmed, except for the association with burning mouth syndrome. This latter result was also surfaced in the Hungarian OHIP study [17]. Possibly, a question about a burning sensation in the mouth is too non-specific to distinguish between patients who do and those who do not suffer from burning mouth syndrome. As in most previous studies, convergent validity, as indicated by the questions about the subjects' self-reported oral health status, and the complaints-related disability, was high. Interestingly, this latter measure, which was newly introduced in the present study, was highly correlated with oral health-related quality of life. This supports their association with a common concept, indicating the influence of oral conditions on a person's daily functioning. Also the group validity was high: the questions about specific complaints related to denture or missing teeth yielded a highly significant relation with the total OHIP-NL scores, showing that problems with oral structures may have a large influence on a person's quality of life indeed. This finding is strengthened by the fact that no relationship was found between the control measures (i.e., the self-reported oral habits) and the total OHIP-NL scores.

## Conclusion

The OHIP-NL offers patients a possibility to describe in detail how their dental problems affect their daily lives. Like the original OHIP-E and its translated versions, the OHIP-NL appears to be a reliable and valid instrument to measure oral health-related quality of life. This makes the instrument a good tool for comparison of this important variable between different countries and cultures.

## Authors' contributions

MM carried out the study and wrote the major part of the paper. MJ initiated the study and did the statistical analyses. MN and FL contributed significantly to improve the design and interpretations of this manuscript. All authors read and approved the final manuscript.

## Pre-publication history

The pre-publication history for this paper can be accessed here:


